# Synthesis of Racemic β-Chamigrene, a Spiro[5.5]undecane Sesquiterpene

**DOI:** 10.3390/molecules191220664

**Published:** 2014-12-10

**Authors:** Simen Antonsen, Lars Skattebøl, Yngve Stenstrøm

**Affiliations:** 1 IKBM, P.O. Box 5003, NMBU, The Norwegian University of Life Sciences, N-1430 Ås, Norway; 2 Department of Chemistry, University of Oslo, P.O. Box 1033, Blindern, N-0315 Oslo, Norway

**Keywords:** natural product, sesquiterpene, β-chamigrene, Diels-Alder, microwave synthesis, spiro[5.5]undecane

## Abstract

The present paper describes a total synthesis of racemic β-chamigrene (**1**), a sesquiterpene with a spiro[5.5]undecane carbon framework. Compared with previously reported β-chamigrene syntheses, we were able to reduce the total number of reaction steps, which also resulted in a significant improvement of the overall yield. The commercially available ketone 6-methylhept-5-en-2-one was transformed by known simple procedures into 3,3-dimethyl-2-methylenecyclohexanone. This reacted with isoprene by a Diels-Alder reaction to give a spiro ketone. An olefination reaction on this compound gave the target molecule.

## 1. Introduction

Chamigrenes are sesquiterpenes containing a spiro[5.5]undecane carbon framework. These terpenes have been isolated from both terrestrial plants and marine organisms [1].

Since the first reported isolation of β-chamigrene in 1967 by Ito *et al.* [2], and the following year by Otha and Hirose [3], several chamigrenes have been isolated almost exclusively from marine sources. In fact more than 120 compounds with the chamigrene carbon frame have, thus far, been isolated from red algae of the genus *Laurencia* and sea hares grazing on them of which many contain chlorine or bromine atoms [1].

Several of the halogenated chamigrenes exhibit interesting antibiotic effect on both Gram-positive and Gram-negative bacteria [4], Furthermore, some chamigrenes, isolated on the Canary Islands in 2005, exhibited cytotoxic activity on HeLa and Hep-2 cancer cell lines [5].

**Figure 1:**
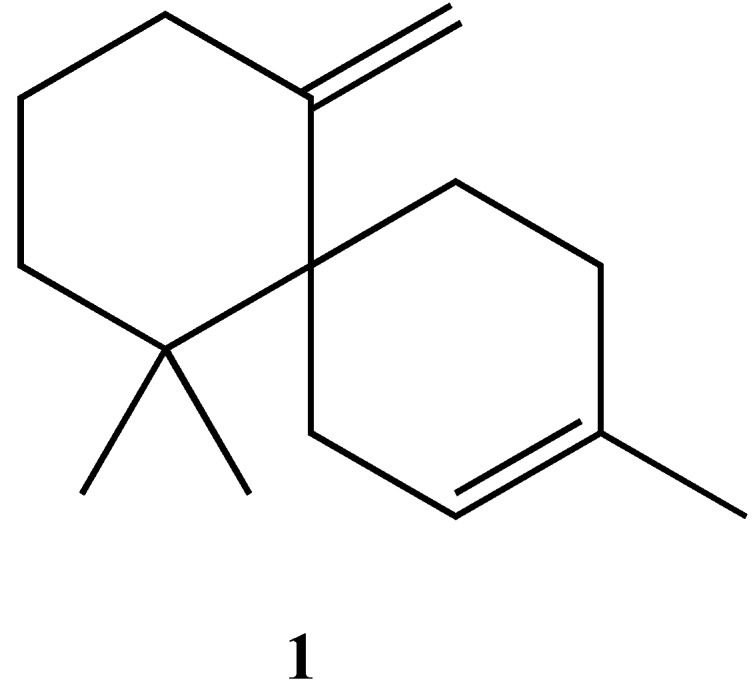
The structure of β-chamigrene.

As a result of the promising biological results, improved syntheses of chamigrenes are still needed. The challenge in synthesizing β-chamigrene is to construct the two quaternary carbon centers. Previously reported total syntheses of β-chamigrene, have addressed this problem using different strategies [1,6]. Based on this knowledge we have achieved a reliable and easy synthesis of β-chamigrene (Figure 1). We anticipate that the same strategy, with some minor modifications, can easily be used for the syntheses of other chamigrenes as well.

## 2. Results and Discussion

**Scheme 1:**
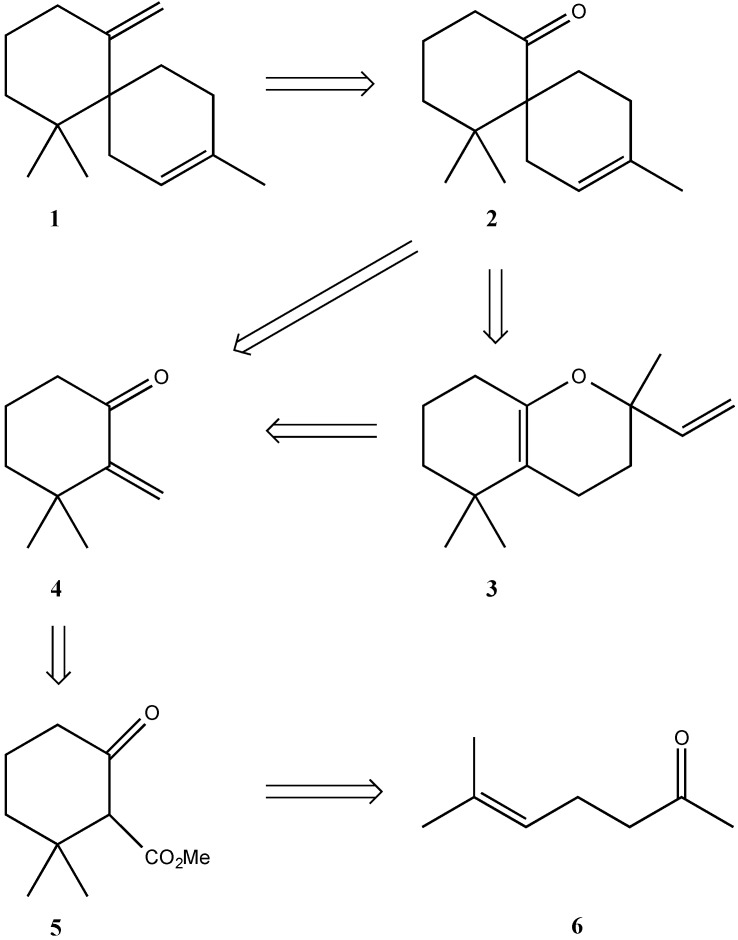
Retrosynthetic analysis of the target molecule **1**.

The retrosynthetic analysis is outlined in Scheme 1. It was anticipated that the spiroketone **2** would be formed either by a direct Diels-Alder reaction on the alkene moiety of **4** or by a Claisen rearrangement of the bicyclic compound **3**, derived from the cyclohexanone **4** and isoprene in a hetero Diels-Alder reaction. Compound **4** has been prepared from the ketoester **5** by Adams *et al.* [6] Following a literature procedure [7] **5** was prepared from the commercially available ketone **6** in 60% yield. According to Adams, both ester and keto functions were reduced to the corresponding diol and subsequently the secondary hydroxyl group selectively protected as the tosylate. In our hands the tosylation reaction always gave white crystals and not an oil, as reported by Adams. A Jones oxidation was employed to reform the ketone and elimination of the tosylate group gave the enone **4** in practically quantitative yield.

Regarding the next step, Ireland *et al.* [8] reported good yields on a similar Diels-Alder reaction using dimethylaluminum chloride as catalyst. When using this catalyst isoprene reacted with **4** at room temperature yielding the spiroketone **2** in 90% yield. The reaction needs four hours to complete, and we tried microwave heating for comparison. Heating **4** and isoprene for 15 min at 160 °C a disappointing 68% yield of the spiro ketone **2** was obtained; however, both conditions compare favorably with the 20% yield reported by Tanaka and co-workers [9]

As mentioned above, formation of **2** can either be a one- or two-step reaction as indicated in Scheme 1. However, the only product isolated was **2**. No further studies to reveal the true mechanism of the reaction were done. 

We also tried other substrates including ethyl methacrylate, methacrylaldehyde and methacrylonitrile for the hetero Diels-Alder reaction of the enone **4** using microwave conditions. Without catalyst, the former, gave the hetero-Diels-Alder product in 77% yield with self-condensation product of ethyl methacrylate as a significant byproduct, while the latter two compounds mainly gave self-condensation products; little or no hetero Diels-Alder adducts were detectable even using a large excess of the dienophiles. Similar results were seen when the catalyst was added. We also tried to preheat the reagents hoping that this would favor the crossed hetero Diels-Alder reaction; however, we still observed mostly self-condensation and little or nothing of the anticipated product. 

**Scheme 2:**
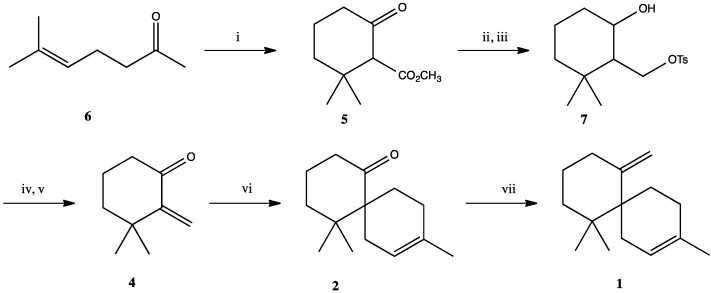
Synthesis of β-chamigrene (**1**).

As reported by Tanaka and co-workers [9], β-chamigrene (**1**) has been obtained from **2** by a Wittig olefination in 70% yield after 48 h at 55–60 °C. A similar Wittig olefination was carried out by Adams *et al.* [2] at 70 °C for 30 h giving the desired final product in only 30% yield. We tried the same Wittig reaction using microwave heating in order to reduce the reaction time, but we were not able to get a yield similar to that reported by Tanaka. In attempts to improve the olefination reaction other methods were tried, including the Peterson [10] and Petasis olefinations [11,12], however, neither of them resulted in improved yields. Finally, we tried a protocol described by Tu-Hsin Yan and co-workers [13], using CH_2_Cl_2_ as a methylene donor promoted by Mg/TiCl_4_/THF. We did a few modifications including the use of sonication and activating the Mg powder by heating it for two hours in the same way that had previously been described for Zn [14]. This procedure was quite facile and gave the target compound **1** in 56% yield with shorter reaction time compared to other methods. The final reaction pathway is showed in Scheme 2. 

*Reagents and Conditions:* (i) Reference [7], 60%; (ii) LiAlH_4_, Et_2_O; (iii) p-TosCl, pyridine, 0 °C, 61% from **5**; (iv) Jones oxidation; (v) DBU, benzene, 2 h room temperature; 100% from **7**; (vi) isoprene, (CH_3_)_2_AlCl (cat), DMC, starting at −10 °C heating to room temperature, 4 h, 90%; (vii) Mg, TiCl_4_, DMC, THF, sonication, 0 °C, 5 h 56%.

## 3. Experimental Section 

All reactions were carried out under N_2_-atmosphere. All reactions were monitored with TLC. The NMR-spectra are recorded on Varian Gemini 300 (300 MHz, Varian Inc., Palo Alto, CA, USA) or Bruker Ascend^TM^ 400 (400 MHz, Bruker Corporation, Billerica, MA, USA). IR spectra were recorded on PerkinElmer FT-IR system Spectrum Bx, 50/60 Hz (PerkinElmer, Waltham, MA, USA). Melting points are recorded on Electrothermal Melting Point Apparatus Model 9100 (Bibby Scientific Limited, Stone, Staffordshire, UK). The GC used to check purity on target molecule was Thermo Finnigan Trace GC (Thermo Electron Corporation, Waltham, MA, USA). The sonication bath was a Branson 5510 (Branson, Danbury, CT, USA). Microwave oven was a Milestone Start synth (132536) (Milestone, Shelton, CT, USA).

### 3.1. 2-Carbomethoxy-3,3-Dimethylcyclohexanon (**5**)

The compound was prepared in 60% overall yield essentially as described by White *et al.* [7], in two steps from the commercially available ketone **6**. The spectral data were identical with those published.

### 3.2. 3,3-Dimethyl-2-Tosyloxymethylcyclohexanol (**7**)

To a stirred mixture of LiAlH_4_ powder (10.7 g, 0.28 mol) in diethyl ether (150 mL) was added dropwise a solution of the keto-ester **5** (13.1 g, 0.07 mol) in 250 mL of diethyl ether The reaction was stirred for an additional hour and quenched at 0 °C by adding 20% aqueous KOH (10 mL), and water. The reaction mixture was stirred for an additional 15 min and filtered. The solid paste was twice triturated with diethyl ether. The organic extract was dried (MgSO_4_), filtered and *conc. in vacuo* to provide 8.9 g of crude diol. This was dissolved in pyridine (200 mL), cooled to 0 °C and *p*-toluenesulfonyl chloride (11.7 g, 0.056 mol) was added with stirring. The reaction mixture was left stirring at 0 °C while monitored on TLC. When all substrate was converted, diethyl ether was added and the solution washed successively with 10% HCl and brine. The organic solution was dried (MgSO_4_) and concentrated *in vacuo* to provide a crude oil, which was purified by flash chromatography (SiO_2_; ethyl acetate:hexane 1:4, R_f_: 0.11) to afford white crystals of tosylate **7** (13.5 g, 61% yield over both steps); Mp. 69–71 °C; ^1^H-NMR (400 MHz, CDCl_3_) δ 0.87 (s, 3H), 0.89 (s, 3H), 1.00–2.00 (m, 7H), 2.43 (s, 3H), 4.06 (m, 1H) 4.14 (dd, 1H, *J* = 9.4 Hz, 3.9 Hz), 4.31 (t, 1H, *J* = 9.4 Hz), (m, 3H), 7.33 (d, 2H, *J* = 8 Hz), 7.78 (d, 2H, *J* = 8 Hz). ^13^C-NMR (100 MHz, CDCl_3_) δ 17.13, 21.68, 23.72, 31.06, 32.40, 32.86, 40.62, 48.66, 65.75, 68.82, 127.88, 129.88, 133.12, 144.79. IR: 3558, 2933 cm^−1^. The spectra data are essentially in agreement with those published [6].

### 3.3. 3,3-Dimethyl-2-Methylenecyclohexanone (**4**)

A solution of tosylate **7** (10.0 g, 0.032 mol) in acetone was treated with excess of Jones reagent (100 mL). The solution was stirred at room temperature for 30 min and the acetone was evaporated. The residue was diluted with water and extracted with diethyl ether. The combined organic extracts were dried (MgSO_4_) and concentrated *in vacuo* to give the keto-tosylate (9.9 g,) that were used in the next step without further purification. A solution of this in dry benzene (200 mL) and DBU (4.6 mL, 0.031 mol) was stirred for 2 h at room temperature. The reaction mixture was washed with water and the organic phase dried (MgSO_4_). Evaporation of the solvents gave the methylenecyclohexanone **4** (4.45 g, quantitative yield) over the two steps ^1^H-NMR (400 MHz, CDCl_3_) δ 1.09 (s, 6H), 1.55–1.65 (m, 2H), 2.75–2.95 (m, 2H), 2.40 (t, 2H, *J* = 13.6 Hz), 5.10 (d, 1H, *J* = 1.3 Hz), 5.63 (d, 1H, *J* = 1.3 Hz). ^13^C-NMR (100 MHz, CDCl_3_) δ 19.80, 20.90, 22.69, 28.46, 29.65, 37.84, 38.59, 40.74, 116.14, 155.74, 203.90. IR (film): 3097, 2961, 1690 cm^−1^. The spectra data are essentially in agreement with those published [6].

### 3.4. 5,5,9-Trimethylspiro[5.5]undec-8-en-1-one (**2**)

A solution of the enone **4** (3.80 g, 0.028 mol) and isoprene (8.5 mL, 0.085 mol) in dichloromethane (20 mL) was cooled to −10 °C with stirring. A 25% solution of dimethylaluminum chloride in hexane (0.034 mol, 19 mL) was added over five min. The solution was warmed to room temperature and stirred for 4 h. The reaction mixture was poured into 100 mL of ice water and the resulting mixture was extracted with diethyl ether. The organic extracts were combined, washed with brine and dried (MgSO_4_). Evaporation of the solvents gave a residue that was purified by flash chromatography (SiO_2_; 1:4 EtOAc:hexane, R_f_: 0.63) to provide the spiro ketone **2** (5.19 g; 90%) as a colorless oil. ^1^H-NMR (400 MHz, CDCl_3_) δ 0.82 (s, 3 H), 0.98 (s, 3 H), 1.35 (dm, 1H, *J* = 14.0 Hz), 1.61 (s (br), 3 H), 1.7 (m, 2 H), 1.83 (m, 1 H), 1.85–1.90 (m, 3 H), 2.04 (td, 1 H, *J* = 13.3 Hz, 4.6 Hz), 2.20–2.26 (m, 2 H), 2.34 (dm, 1 H, *J* = 2.0 Hz, 3.5Hz), 2.65 (dt, 1H, *J* = 12.8Hz, 6.0Hz), 5.36 (d (br), 1H, *J* = 3.5 Hz). ^13^C-NMR (100 MHz, CDCl_3_) 23.03, 23.19, 23.21, 24.79, 26.31, 27.02, 27.72, 35.52, 37.02, 40.77, 54.85, 120.44, 131.69, 214.88. IR (film): 2960, 1731 cm^−1^. The spectral data are in agreement with those published.

### 3.5. 5,5,9-Trimethylspiro[5.5]undec-8-en-1-one (**2**) (With Microwave Heating)

A solution of the enone **4** (1.40 g, 10 mmol) and isoprene (7 mL, 70 mmol) was cooled to −10 °C and a 25% solution of dimethylaluminum chloride in hexane (7 mL, 13 mmol,) was added over 5 min. The solution was transferred to a microwave reactor, flushed with N_2_ and reacted for 15 min. at 160 °C. The reaction mixture was cooled to room temperature and poured into ice-water (100 mL). The resulting mixture was extracted with diethyl ether, and the extract washed with brine and dried (MgSO_4_). Evaporation of the solvent gave an oil that was purified by flash chromatography as described above to give the spiro ketone **2** (1.41 g; 68%) as colorless oil. 

### 3.6. 3,7,7-Trimethyl-11-Methylenespiro[5.5]undec-2-ene (**1**)

Mg powder (0.192 g, 8 mmol) was heated to 160 °C for 2 h with vigorous stirring under N_2_ before being cooled to 0 °C in a sonication bath [14]. A solution of TiCl_4_ (0.21 mL, 2 mmol) in dry CH_2_Cl_2_ (4 mL) was then added. A solution of the spiroketone **2** (0.206 g, 1.0 mmol) in dichloromethane (3 mL) and THF (2 mL) was added dropwise to the cooled Mg/TiCl_4_/CH_2_Cl_2_ mixture. The reaction mixture turned green/black when the addition was completed. The mixture was kept at 0 °C for 30 min. before slowly heating it to reflux. The reaction was kept at this temperature with sonication for an additional 5 h, cooled to 0 °C and saturated aq. K_2_CO_3_-solution (10 mL) was added. The solution was diluted with diethyl ether and the organic phase was separated, dried (MgSO_4_) and the solvent was carefully distilled off under N_2_-atmosphere to give a yellow oil. This oil was purified by chromatography (SiO_2_, pentane, R_f_ = 0.70). to provide β-chamigrene (**1**), (0.11 g, 56%) as a colorless oil, pure by GLC. ^1^H-NMR (400 MHz, CDCl_3_) δ 0.84 (s, 3H), 0.89 (s, 3H), 1.15 (dm, 1H, *J* = 13.5 Hz) 1.25–1.35 (m, 1H), 1.45–1.55 (m, 1H), 1.59 (s (br), 3 H), 1.65–1.85 (m, 4H), 1.90–2.20 (m, 4H), 2.25 (td, 1H, *J* = 13.1 Hz, 5.5 Hz), 4.54 (s (br),1H), 4.89 (s (br), 1H) 5.32 (d (br), 1H, *J* = 2.3 Hz). ^13^C-NMR (100 MHz, CDCl_3_) δ 23.06, 23.84, 25.03, 25.99, 27.92, 28.99, 29.72, 32.23, 37.02, 37.27, 44.76, 110.52, 120.01, 132.73, 149.06. IR (film): 3074, 2956, 2928, 1638, 1454 cm^−1^. Spectral data are in agreement with those published [1].

## 4. Conclusions

In summary, we have accomplished a simple and reliable total synthesis giving β-chamigrene in a total yield of 18.4% from inexpensive starting materials. 
