# Erratum: Antonsen, S.; et al. Synthesis of Racemic β-Chamigrene, a Spiro[5.5]undecane Sequiterpene. *Molecules* 2014, *19*, 20664–20670

**DOI:** 10.3390/molecules23030704

**Published:** 2018-03-20

**Authors:** Molecules Editorial Office

**Affiliations:** MDPI AG, St. Alban-Anlage 66, 4052 Basel, Switzerland; Tel.: +41-61-683-77-34; Fax: +41-61-302-89-18

The Molecules Editorial Office wishes to make the following erratum to this paper [1]. There is a misspelling in the title of “Synthesis of Racemic β-Chamigrene, a Spiro[[5.5]undecane Sequiterpene”. The correct title should be “Synthesis of Racemic β-Chamigrene, a Spiro[[5.5]undecane Sesquiterpene”.

The change does not affect the scientific results. The manuscript will be updated and the original will remain online on the article webpage. We apologize for any inconvenience caused to the readers by this mistake. 
